# Book review: FLIES – The Natural History and Diversity of Diptera Stephen A. Marshall (2012) Firefly Press Ltd., 616 pp.

**DOI:** 10.3897/zookeys.261.4628

**Published:** 2013-01-24

**Authors:** Terry L. Erwin

**Affiliations:** 1 Hyper-diversity Group, Department of Entomology, MRC-187, National Museum of Natural History, Smithsonian Institution, P.O. Box 37012, Washington D.C., 20013-7012, U.S.A.

*Human knowledge will be erased from the world’s archives before we possess the last word that a gnat has to say to us.*

-Henri Fabre-

The new book, FLIES, tells us a lot about what gnats and their relatives have to tell us about nature, would we but listen attentively.

**Figure:**
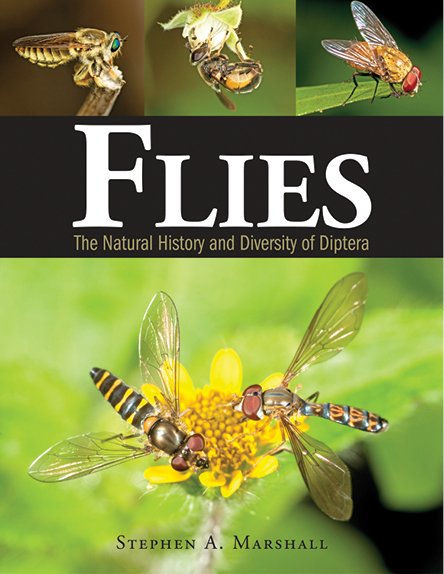


The author, Stephen Marshall, began his biological interests in beetles and then somehow went to the dark side, FLIES. This reviewer began his career in beetles and stayed put; that is what qualifies him as a reviewer of this simply superb book Steve Marshall has produced both in text and images. Why? It’s in the subtitle: the Natural History and Diversity of Diptera, the key word being ***diversity*** by which Marshall means many different kinds. Beetles and parasitic wasps are universally touted as the hyper-diverse and divergent groups of insects, so why does Marshall claim that Flies also exhibit such diversity in the natural world? In doing so, he sets himself apart from what more focused Dipterists have usually done, that is stay the course, look at one genus, one family, one region at a time; not much diversity displayed on their watch, for sure. Marshall, on the other hand, took on the entire dipteran Order, globally, with hundreds of superb photos (adults and immatures) that he took in the field over years of searching out fly species richness. He added an up-to-date text covering all taxa of the entire Order at the subfamily and tribal levels with hundreds of examples and images named at the genus and/or species level.

Marshall’s FLIES is written and imaged for both the academic and the enthusiastic layperson. It contains some 2000 color images of flies in their natural environment, nearly all taken by the author. He has selected (out of some 160,000 named species of flies), those that he finds especially fascinating and those essential for demonstrating global dipteran diversity in form and function. The latter, *form and function*, speak directly to the astounding diversity and divergence of dipterans and justify including them along with the celebrated richness and abundance of beetles and parasitic wasps. Marshall, in FLIES, has justified that leap with his broad coverage and excellent story telling about flies’ lives.

Such dipteran diversity is a mixed blessing for the public and for scientists alike. Who wants to see a blow fly from your neighbor’s garbage bin walking around on your chocolate cake, or a dozen mosquitos landing on you when you are trying patiently to photograph a hummingbird at a tropical flower in an Amazonian *Heliconia* patch? On the other hand, colorful hover flies on a yellow flower (Marshall’s book cover jacket) are part of nature’s exquisite beauty. While many groups of flies are vectors of devastating human and animal diseases and pests of crops and forests, others are important pollinators, recyclers, and beneficial control agents of insect pests. Marshall’s text tells compelling stories about all these kinds of flies that are easily read by citizen naturalists and high-schoolers in science class, as well as professional biologists of all walks of study.

While Marshall’s introduction claims that “flies rule!” and his book sets out to prove that claim, Coleopterists and Hymenopterists certainly will argue that point. However, this reviewer has to agree that in the Amazon Basin during the rainy season there is more fly abundance by far in the rain forest canopy than any other group of insects (except relatively species-poor but abundant ants). This is thanks to one family of dipterans, the fungus gnats, Mycetophilidae (see Marshall’s pages 139-140) that emerge by the multimillions, as fungi reach their fruiting peak. While flies might not truly “rule,” gnats do have a lot to say about temperate and tropical fly abundance, something that Henri Fabre would appreciate.

Without a doubt, Marshall sets high standards with this and his previous book, *Insects: Their Natural History and Diversity*, (also, Firefly Books). These treatments offer a challenge to entomologists of other ordinal taxa to produce similar books. Should that come to pass, and considering that insect and their relatives constitute three-fourths of life on earth, we humans would have a far better understanding of the natural world and our place in it.

